# Gaps in Hospice and Palliative Care Research: A Scoping Review of the North American Literature

**DOI:** 10.1155/2020/3921245

**Published:** 2020-11-05

**Authors:** Rebecca Antonacci, Carol Barrie, Sharon Baxter, Sarah Chaffey, Srini Chary, Pamela Grassau, Chad Hammond, Mehrnoush Mirhosseini, Raza M. Mirza, Kate Murzin, Christopher A. Klinger

**Affiliations:** ^1^Institute for Life Course and Aging, Factor-Inwentash Faculty of Social Work, University of Toronto, 246 Bloor Street West, Suite 238, Toronto, Ontario M5S 1V4, Canada; ^2^National Initiative for the Care of the Elderly, 246 Bloor Street West, Suite 234, Toronto, Ontario M4S 1V4, Canada; ^3^Canadian Frailty Network, Kidd House, 100 Stuart Street, Kingston, Ontario K7L 3N6, Canada; ^4^Quality End-of-Life Care Coalition of Canada, Annex D, Saint-Vincent Hospital, 60 Cambridge Street North, Ottawa, Ontario K1R 7A5, Canada; ^5^Canadian Hospice Palliative Care Association, Annex D, Saint-Vincent Hospital, 60 Cambridge Street North, Ottawa, Ontario K1R 7A5, Canada; ^6^Pallium Canada, 75 Bruyère Street, Ottawa, Ontario K1N 5C7, Canada; ^7^School of Social Work, Carleton University, Dunton Tower, 1125 Colonel By Drive, Suite 509, Ottawa, Ontario K1S 5B6, Canada; ^8^Faculty of Medicine and Dentistry, University of Alberta, College Plaza, 112 Street, Suite 205, Edmonton, Alberta T6G 2R3, Canada; ^9^Realize, 1240 Bay Street, Suite 600, Toronto, Ontario M5R 2A7, Canada

## Abstract

**Background:**

The demand for hospice and palliative care is growing as a result of the increase of an aging population, which is most prominent in North America. Despite the importance of the topic and an increase in hospice and palliative care utilization, there still are gaps in research and evidence within the field.

**Aim:**

To determine what gaps currently exist in hospice and palliative/end-of-life care research within the context of a North American setting to ensure that future directions are grounded in appropriate evidence.

**Methods:**

Using Arksey and O'Malley's scoping review framework, six peer-reviewed, and four grey electronic literature databases in healthcare and the social sciences were searched in mid-2019. 111 full-text articles were retrieved, with 25 articles and reports meeting the inclusion criteria. Major themes were identified through thematic context analysis: (1) clinical, (2) system access to care, (3) research methodology, and (4) caregiving-related research gaps.

**Results:**

Findings include strategies for engaging stakeholder organizations and funding agencies, implications for other stakeholder groups such as clinicians and researchers, and highlight implications for policy (e.g., national framework discussion) and practice (e.g., healthcare provider education and training and public awareness).

**Conclusion:**

Reviewing and addressing targeted research gaps is essential to inform future directions in Canada and beyond.

## 1. Background

The increase of an aging population is happening all over the world but has become most prominent in North America [1]. As a result, the demand for hospice and palliative care (HPC) is growing [1]. In the Canadian context, a 1999 report by the Canadian Palliative Care Association (CPCA, now CHPCA) identified that Canadians were concerned about the type of care they would receive when they died [2]. Since then, there has been a shift with new advances in the end-of-life care (EoL) field. For example, a national advance care planning (ACP) strategy for Canada was developed in 2013, The Way Forward initiative [3, 4] created a framework for an integrated palliative approach to care, in 2016, Bill C-14 was passed, which legalized medical assistance in dying (MAiD) [5], and in 2018, Health Canada introduced a new Framework on Palliative Care in Canada [6]. Therefore, it is important to have as much information about HPC available so that individuals and decision makers in North America can reach an informed, evidence-based choice for the end of life.

In the 1999 CPCA report, it was expressed that there was a lack of research in the field of HPC, and still currently, in the absence of a national minimum data set (MDS) and national standards, there is also little information available at a population level about how care is delivered in different provinces and territories and how it is utilized in different healthcare settings across Canada [2, 7, 8]. This may lead to gaps in areas of HPC that—if remedied—could improve the field and the usage of specific EoL services [8]. Therefore, it is important to understand what gaps exist within HPC/EoL in order to direct future research and funding to improve this field of care.

Following the completion of the Canadian Institutes of Health Research's (CIHR) first Palliative and End-of-Life Care Initiative [9] and building on a previous environmental scan on HPC research funding [7], the Quality End-of-Life Care Coalition of Canada (QELCCC) commissioned a scoping review of the North American literature to identify the gaps that need to be addressed moving forward with HPC and EoL research.

The research question was as follows: “What gaps currently exist in HPC/EoL research in Canada/the North American context as outlined in the literature?”

This paper will also address several themes that were discovered from the literature using thematic content analysis [10]. These themes were as follows: clinical elements of HPC, system access to care, research methodology, and experiences of caregivers.

## 2. Methods

A review of the North American literature based on Arksey and O'Malley's framework was conducted to analyze gaps in HPC/EoL research [11]. Arksey and O'Malley identified a scoping review as addressing broader research questions rather than focusing on a specific point, allowing for more breadth of literature to be included [11]. Their framework is comprised of the following steps: (1) identifying the research question, (2) identifying relevant studies, (3) study selection, (4) charting the data, and (5) collating, summarizing, and reporting results [11]. Each phase will be described in further detail below.

### 2.1. Data Sources, Search Strategy, and Selection

The electronic databases CINAHL, Embase, Medline (Ageline), ProQuest Dissertation and Theses, PubMed, and Social Work Abstracts were searched using a variety of key terms/medical subject headings (MeSH) such as “aged”, “hospice”, “palliative care”, and “research gaps” alongside search strings employing Boolean operators (e.g., AND, OR, and NOT). Further details can be found under Table 1. Grey literature sources were also searched using major North American stakeholder organizations such as the American Heart Association, Canadian Cancer Society, Canadian Institutes of Health Research, Canadian Hospice Palliative Care Association, National Hospice Foundation, National Hospice Palliative Care Organization, hand search, and greylit.org (Table 1).

### 2.2. Selection Criteria

**Figure 1:**
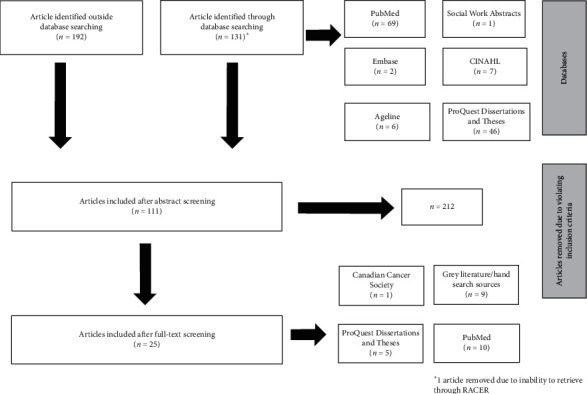
Search strategy.

Any type of empirical study (qualitative/quantitative; randomized controlled trial, case study, review, and so on) was included, provided it met the following criteria: a focus on hospice and palliative care for populations aged 65+, relevance to the North American context (European studies were included if they could be mapped on to a North American context, i.e., results related to North America), published in English between the years 2000 and 2019 (there were very few studies published prior to 2000, and these were likely picked up by the 1999 CPCA report on this topic) [2], and referencing research/knowledge gaps and/or discussing future steps for research in the field of HPC/EoL. Both papers that were written to highlight research gaps and those that discussed specific gaps related to their research question were included. Exclusion criteria included studies that did not explicitly address hospice and palliative care (e.g., papers identifying research gaps related to the diagnosis and biomedical treatment of specific diseases), those focused on pediatric hospice and palliative care (as this is a special population that would require a separate review), papers without reference to research or knowledge gaps, studies deemed not relevant to the North American context, and those not published in English. Unfortunately, we were not able to include Canadian studies published in French as we did not have ready access to a translator. Each study was reviewed to determine whether it satisfied inclusion criteria (options: yes, no, and maybe) by two reviewers (RA and SC) independently. A third reviewer (CAK) read the studies placed in the “maybe” category and broke any ties. Multiple reviewers were used in order to maintain scientific rigor and to ensure that studies selected were in line with the central research question posed. The selection process is outlined in Figure 1.

### 2.3. Data Charting

A data extraction table was created, which outlined the data to be collected from each study (Table 1). The first author went through the final list of articles and extracted the following information: (1) author(s), (2) year of publication, (3) title of publication and journal/publication source, (4) study design and study objective, (5) geographic region, (6) findings, (7) themes, and (8) database/retrieval source.

## 3. Results

From 2,500+ articles identified through the literature search, 131 were included from database searches and 192 from non-database/hand searches for initial screening. From the database searches, 1 article was included from Social Work Abstracts, 2 from Embase, 6 from Ageline, 7 from CINAHL, 46 from ProQuest Dissertation and Theses, and 69 from PubMed. We selected 111 sources based on screening the abstracts identified through the database and hand searches. Following a full-text review, 1 article was included from the Canadian Cancer Society, 5 from ProQuest Dissertations and Theses, 9 from grey literature/hand searches, and 10 from PubMed. From these final 25 articles, utilizing thematic content analysis [10], parent themes were identified, and upon reading abstracts and full-texts, subthemes were identified by the reviewers (RA, SC, and CAK) [11]. Themes were selected after reading each article and taking note of common results and research questions outlined in each paper. Following discussion with the research team, each study was grouped by the following common themes pertaining to gaps in HPC/EoL research: (1) access to care with the subthemes of location (rural and urban), and socioeconomic status and minority cultures, (2) clinical with the subthemes of cultural sensitivity, grief and bereavement, and education and training for healthcare workers, (3) caregiving, and (4) research methodology with the subthemes of operationalization of terms, data availability, and study designs (Table 1).

### 3.1. Access to Care

Access to care appeared as a theme in 32% of articles (*n* = 8) [12, 17, 22, 23, 25, 26, 33, 36]. One initial gap in access to care was that the EoL system did not adequately address the needs of those with serious or chronic illness(es) but was rather more focused on acute care [22]. As well, there was a lack of HPC/EoL research in long-term care facilities and (residential) hospices. Additionally, older adults and multimorbid individuals (having multiple health conditions) [25] were often excluded from clinical trials on improving access to care.

#### 3.1.1. Minority Groups

Another population which faces a gap in their ability to access HPC/EoL is those of certain cultural or ethnic groups. 16% of papers (*n* = 4) discussed the subtheme of minority groups [12, 17, 26, 33]. Two studies indicated that not all EoL was accessible to every population as not all EoL was culturally relevant or sensitive [12, 26]. In a study that looked particularly at the under-represented cultural group of Latinos in the United States, the authors found that this group experienced a gap in culturally appropriate care especially when trying to access care within the public healthcare system [17]. This study suggested that one way to potentially address this gap was to have more targeted advocacy efforts towards culturally sensitive elements of care (to be identified) [17]. Specifically, in Canada, one of the needs pertaining to access to culturally relevant HPC/EoL is among Indigenous Peoples [33]. For example, in a document by the Six Nations of the Grand River Territory, they identified a need to create and to develop (research) policies with the input and collaboration of both healthcare providers and members of Indigenous communities [33].

#### 3.1.2. Access to Care Based on Affordability and Location

Three papers (12%) discussed access to care based on affordability and location [12, 23, 36]. There was a lack of information on cost-effective care to ensure that all members of the population had access regardless of their socioeconomic status and also access to community-based supportive care resources [36]. Moreover, there was a disconnect between actual and preferred place of death which resulted from little to no community-based care [12, 23]. Thus, there was a gap in understanding the care needs of those who died in other places besides hospitals, such as in the community or at home. Therefore, more data were needed on the experiences of individuals (and their family members) in residential (including hospice) and home care settings alongside marginalized communities/populations [36].

### 3.2. Clinical

Studies discussing research gaps under the clinical theme comprised 56% of all papers found (*n* = 14) [12, 15, 17–21, 23, 26, 27, 30, 31, 33, 35].

#### 3.2.1. Cultural Sensitivity

The subtheme of culturally sensitive care also overlapped in papers containing the clinical theme. These overlaps were particularly evident in five papers (20%) [12, 17, 26, 30, 33], one of which highlighted a need for the development of assessment tools that were culturally appropriate for diverse groups [30]. For example, this might include pain scales in multiple languages to be more easily understood by the patient [30]. Additionally, healthcare providers might want to consider presenting information in a culturally appropriate way, for example, using the native language or considering alternative treatments to Western medicine [12, 17]. As well, part of the way that this gap in providing culturally sensitive care could be addressed is to offer training that touches on topics related to providing culturally safe and relevant treatment for those in the healthcare field [33]. Finally, there was a need for more ethnocultural training models to address how cultural factors may impact outcomes for those in HPC [26].

#### 3.2.2. Training and Education of Healthcare Providers

Another gap in the clinical elements of HPC/EoL pertains to the subtheme of overall training and education that healthcare providers and palliative healthcare providers received (32%, *n* = 8) [12, 15, 18–20, 23, 27, 35]. For example, five studies reported that healthcare providers expressed uncertainty in communicating with patients as they experienced gaps in knowledge, skills, and training [12, 15, 18, 31, 35]. As well, there were differences in the amount of HPC training each healthcare provider received, with some having more knowledge on end-of-life issues than others [15, 23, 27]. Healthcare providers felt that the gaps in education and training limited their ability to communicate with patients. Specifically, in relation to nurses, it was found that there was a gap in their awareness of patient needs in decision-making that warrants further research [19]. Additionally, there needs to be more training to deal with patients of different ages such as older individuals who need HPC services [20]. There was also a desire for more tools and training programs that can help to address the gap between desired and actual decision-making in HPC/EoL patients [19].

#### 3.2.3. Grief and Bereavement

Last, 1 paper (4%) addressed gaps that existed within the subtheme pertaining to practitioners' understanding of grief and bereavement such as the grief process including growth and resilience, how to differentiate grief from a mental illness, and how to refer those grieving to the appropriate services [21]. As well, there was a lack of research into EoL decision-making and the relationship between advance care planning and advance directives with grief [21].

### 3.3. Caregiving

Caregiving was a theme in 32% of papers (*n* = 8) [12, 16, 21, 23, 28, 31, 34, 35], and the literature also revealed research gaps in terms of support for caregivers of those in EoL. It was found that gaps existed in mental health and emotional support for caregivers [23, 31]. Moreover, gaps were present in terms of identifying populations of caregivers who needed support and in better understanding the needs of and outcomes for caregivers in general [34, 35]. More specifically, there was a lack of research on caregivers who supported family members with advanced illness and how they were impacted by the caring process [28]. Tying into the theme of research methods, elements of caregivers and caregiving in EoL were assumed rather than directly tested in studies [16]. Thus, gaps existed in understanding through evidence how caregivers prepared for their role and how they adjusted following the death of the individual they were caring for [16]. Moreover, there was little knowledge on how EoL grief impacted family caregivers and the specific relationship between caregiving and grief alongside a lack of bereavement services [12, 21, 31].

### 3.4. Research Methodology

The theme of research methodology appeared in 60% of papers (*n* = 15) and encompassed three subthemes including study design, operationalization of terms, and data availability in studies [12–14, 18, 20, 23–26, 29, 31, 32, 34–36].

#### 3.4.1. Operationalization

Operationalization of terms refers to how studies defined certain constructs relating to EoL such as quality of life or quality of death. 16% of papers contained the subtheme of operationalization (*n* = 4) [20, 25, 31, 35]. Beginning with operationalization, there was a lack of understanding and unity across terms such as quality of life and quality of death, defining the target populations for studies and what made a quality HPC program [31, 35]. For example, there was not a clear definition for who the target population was for HPC research as some studies focused on patients with a serious illness such as cancer, whereas others focused on psychosocial elements of dying [20, 35]. Additionally, when research was performed on patient populations who were multimorbid—meaning they had more than one condition—it was unclear what was included or considered “multimorbid” [25].

#### 3.4.2. Data Availability

Five papers identified gaps in data availability (20%) [12, 23, 34–36]. There were also gaps in the data available for researchers to use, for example, data such as surveillance data were collected infrequently, and data collection overall remained inconsistent and was not standardized in the field of HPC/EoL research [12, 23, 35]. These gaps were also present in a lack of standardized data including death certificates, hospital discharge abstracts, and drug plans [36]. As mentioned previously, caregivers were also left out of many of the clinical elements of palliative care, but they were also excluded as a population in the research as seen in the lack of data on post death interviews and satisfaction with care [34, 35].

#### 3.4.3. Study Designs

Gaps in research study design were present in 10 papers (40%) [12–14, 18, 24, 26, 29, 32, 35, 36]. One initial reason that there may be gaps in how studies dealing with HPC/EoL were designed was as a result of incongruency between the EoL organization's understanding of research and the researchers themselves [32]. Additionally, there was a need for more longitudinal studies, which follow patients across different settings and time periods during their end-of-life journey, and more studies that focus on levels of access to HPC, and who can access it and who cannot (and why) [32]. There was also a need for more studies focusing on the oldest old in EoL (age 80+), interruption in hospice care, both qualitative and quantitative studies that focus on the healthcare preferences, quality of care, appropriateness of dying in a hospital and adequacy of symptom management tools, evaluation of economic elements that go beyond saving money, and also how decision-making is impacted in sudden trajectory situations meaning a sudden change in the trajectory of dying [14, 24, 26, 35, 36]. Last, there was a need for more active and accurate recruitment of patients for research studies, better randomization, and studies that address and refine the practical aspects of care [12, 18, 24, 35]. For example, there was a need for more research into how models developed through research can improve the actual quality of care in a real-life setting. There was also a need for more research into those with advanced dementia, specifically pertaining to the care experience, including families, and also quality of life [35]. Moreover, there was a need for studies that address advance care planning in this population to look at various factors not currently addressed such as the timing of advance care planning conversations, care goals and preferences for substitute decision makers, and also how to document and carry out wishes [35].

## 4. Summary of Results and Discussion

The purpose of this scoping review was to gather an understanding from the current literature about the gaps and needs in HPC/EoL research. There were 25 studies included in the review, and although a majority were from North America, there were some (*n* = 6) included from outside of North America [13, 16, 18, 21, 24, 32] as they could easily be applied to a North American context. Overall, there were limited studies exclusively addressing gaps in palliative care found during the search phase, which suggested that perhaps there was not enough focus in the field of palliative care research on what was needed for improvement. The most common issues addressed by all the papers were gaps in access to care, clinical elements of HPC/EoL, and research methodology. Each of the themes discussed related to gaps that need to be addressed in different areas of research in HPC/EoL. While the theme of clinical gaps remained among the most prominent throughout the articles included (*n* = 14, 56%) [12, 15, 17–21, 23, 26, 27, 30, 31, 33, 35], access to care—specifically access for minority or marginalized populations—was another important topic [12, 17, 26, 33]. Some issues regarding gaps in research methodology were a lack of funding, adequate sample sizes, and data availability. Last, although caregiving was among the themes with the least amount of papers (*n* = 8, 32%) [12, 16, 21, 23, 28, 31, 34, 35], it was still expressed as a gap in research, specifically providing adequate support to caregivers pre death and post death. There is also similar evidence from Taiwan, where a study analyzing preferences in HPC for both patients and caregivers was conducted in response to the lack of literature on caregivers in the HPC field [37]. Additionally, many of the papers that addressed caregiving focused on the lack of support for caregivers in HPC. This theme has clearly remained over time as also highlighted in the 1999 CPCA report [2]. Moreover, many of the other themes have remained the same since the 1999 report up to now (such as clinical decision-making and cultural sensitivity). What has changed, however, was that the 1999 report focused on increasing communication between organizations, whereas now communication was more an issue between patients and practitioners. As well, the 1999 report went beyond a literature search to include focus groups and an analysis of research funding [2]. In this vein, both the QELCCC [7] and the Canadian Cancer Research Alliance [38] have observed an unfortunate decline in research funding in Canada in recent years, with a particular need for seed and proof of concept (without matching) funding [7].

### 4.1. Implications for Research

Although research is actively being conducted on HPC and EoL topics, there are gaps in clinical and health services research alongside the populations included. There specifically is a lack of research on the experience of individuals in HPC within ethnoracial communities, the oldest old (80+), caregivers, and a lack of qualitative and longitudinal studies. This may mean that not all the experiences of those in HPC are considered, and therefore, care models that stem from research may not be appropriate for all types of patients. One way this gap can be remedied is perhaps by establishing/fostering a palliative research network [28, 39]. A research network dedicated specifically to palliative care can also help to refine and establish a clearer agenda of research needs [34]. The new Pan-Canadian Palliative Care Research Collaborative (PCPCRC) might be such a network for Canada [34, 40].

Improving the criteria for those involved in research, such as by adjusting operationalization of terms such as quality of death, multimorbidity, and creating clearer definitions of populations enrolled in research, was also highlighted in the literature [20, 25, 31, 35]. Improvements are already being made in the field that could be used more frequently such as creating broad eligibility criteria which can reduce research burden [38, 41]. As well, the CHPCA developed a lexicon of HPC terms in order to address issues around operationalization and terms used [4]. Consistently screening participants involved in studies to make sure they continue to meet the guidelines set out by the research team can allow for the population being included to be more clearly defined [38, 41]. Additionally, consistently reviewing the research protocol at the beginning of a clinical trial was found to help research teams adjust the protocols easier and to accommodate changes [41]. Moreover, establishing a connection between the clinical team and research team in palliative care units and other settings can help to create uniformity in the studies [38]. Finally, reframing the benefits of research for those who are dying may be helpful for future studies [42]. Those who participated in research cited these benefits as including being able to help others and increase their autonomy through their participation [42].

### 4.2. Implications for Practice

Providing culturally sensitive care was one element of a gap in practice that appeared in quite a few papers [12, 17, 26, 30, 33]. Having a lack of healthcare practitioners available to provide culturally appropriate services means those at the end of life from diverse cultural backgrounds may be uncomfortable with the current palliative services available or may feel dissuaded from using or accessing the EoL available. This may in turn limit their access to quality EoL. Another practice gap identified was the dissonance between levels of training and education in healthcare practitioners [35]. This poses an issue as patients may not receive uniform care across the EoL settings/continuum. Thus, not only standardizing the training procedures of HPC/EoL practitioners but also incorporating EoL training into all healthcare professionals' education can improve the quality of care given to all patients. Last, it was stated in the literature that there was a gap in support for caregivers, and their needs and preferences were often ignored [34]. Caregivers are very much involved with the patient and therefore are impacted by the dying process. Thus, without proper support and needs identification, caregivers may experience mental health issues and grief that they cannot properly resolve. Returning to the topic of culturally sensitive care, caregivers from non-Western backgrounds may also experience problems if they cannot access culturally sensitive follow-up care or support during the EoL process.

### 4.3. Implications for Policy

There are gaps that currently exist between different cultures' needs in HPC/EoL and the care provided. One suggested recommendation for this is to create policies or agreements between service providers and different cultural communities to improve access to care for those in different cultural groups and to highlight a clearer understanding of the roles in both communities for care [33]. One of the gaps addressed in the studies regarding access to care is making EoL services available for those in hard to access communities [12]. One way that this gap could be improved is by formulating guidelines for how care can be accessed in these communities that do not have easy access to hospice and/or palliative care services alongside fostering compassionate communities [33]. Additionally, guidelines addressing funding may also help to appropriately direct the community's finances into creating affordable and accessible community-based care. Much of the information to improve and create new policies exists; however, the issue is that the available data are not translated or standardized yet into a format that can be set in a practical way such as to improve policy (e.g., national MDS) [12].

The studies reviewed also highlighted gaps in levels of training received by those working in palliative care settings and in healthcare/healthcare administration in general [12, 15, 17–19, 26, 27, 31, 33, 35]. In order to standardize and provide the best care for patients, a palliative care competency framework should be considered as guidelines on education for healthcare providers specifically for practitioners dealing with different cultural groups. This and the aforementioned are to be part of the envisioned Framework on Palliative Care in Canada [6].

### 4.4. Limitations

Some limitations of this scoping review were the following: there was no translator readily available to authors; thus, all papers included had to be in English. This means that some papers addressing gaps in HPC research especially from French-speaking Canada may have been left out. Additionally, only 25 articles met the inclusion criteria, which is a relatively small number, and therefore may not address all of the issues in gaps pertaining to HPC/EoL. Furthermore, the focus was restricted to a North American context. Additionally, some articles that related to the theme of this literature review but did not have a full-text available online were requested through RACER but never received (Table 1). RACER is an online library system that can provide full-text articles that are not available online to those who are requesting them. Thus, not all papers pertaining to the topic were included in the final list of articles.

## 5. Conclusions

The field of HPC/EoL is evolving in Canada with new modes of treatment and options for a patient including legal right to medical assistance in dying (MAiD) [5]. It is important to acknowledge the gaps in research and practice in order to improve the field in an appropriate and meaningful way of reducing suffering at the end of life. North American literature pertaining to gaps in HPC is limited suggesting a need for future research to be directed specifically on identifying gaps as perceived by patients, caregivers, and practitioners. The content in each of the themes can provide a basis to inform future funders and researchers about important topics that need to be addressed and can help direct the focal points of future HPC/EoL research and practice in Canada and beyond.

## Figures and Tables

**Table 1:** Data extraction table.

Citation number	Author(s); title of publication; journal	Study design; purpose	Region	Findings	Themes	Database
[12]	Bray et al.; Pan-Canadian Framework for Palliative and End-of-Life Care Research	(i) Report (ii) The purpose of this report was to develop a national framework to guide funders in end-of-life care research	Canada	(i) Need for research support surrounding evidence about improving advance care planning and community-based peer support programs and economic elements of delivering palliative care across different settings (ii) More research into evidence that can improve training programs for healthcare professionals (iii) More research into alternative medications for those who cannot handle standard treatment regimens and also caregiving, cultural, psychosocial, and existential aspects of death (iv) More training for all healthcare providers to deal with palliative patients (v) There is minimal research into evaluating the best training models for healthcare practitioners in palliative care (vi) More interventions into making palliative care accessible for marginalized populations and those in hard to reach populations (vii) There is a need to translate the information gathered in palliative care research into practical elements (practice and policy) and a need to standardize data	Research methodology, clinical, system access to care, and caregiving	Hand search

[13]	Bolmsjo; End-of-Life Care for Old People: A Review of the Literature; American Journal of Hospice and Palliative Medicine	(i) Literature review (ii) Purpose of this review was to highlight inventory elements of end-of-life care for older individuals and to illuminate gaps in knowledge	Sweden	(i) There is a need for more discussion of suffering in different cultures (ii) More in depth research into palliative care, specifically empirical studies to assess who has access to palliative care (iii) More studies needed on the oldest old defined as 80+ (iv) More research needed for family relationships in palliative care	Clinical, research methodology and caregiving	PubMed

[14]	Hendricks; Factors Influencing Interruption in Hospice Home Care	(i) Quantitative (ii) The purpose of this work was to analyze the decision-making process behind why patients leave and then return to hospice care, referred to as interruptions	The United States	(i) Interruption in hospice care is an under-researched area in hospice home care (ii) There is a need for more qualitative inquiry into understanding these interruptions and also to evaluate the result of hospice interruptions to compare how one utilizes a home health aide in comparison with being alone	Research methodology	ProQuest Dissertation and Theses
[15]	Chuang et al.; “I Just Felt Like I Was Stuck in the Middle”: Physician Assistants' Experiences Communicating with Terminally Ill Patients and Their Families in the Acute Care Setting; Journal of Pain and Symptom Management	(i) Qualitative (ii) Purpose of this study was to explore the role physician assistants serve in communicating with terminally ill patients/families	United States	(i) Uncertainty in communicating with patients because of gaps in knowledge, skills, and education (ii) Issues with structural deficits in the patient care system (iii) Recommendations to target institutional structures, hospital systems, and roles and responsibilities	Clinical	PubMed

[16]	Breen; The Effect of Caring on Post-Bereavement Outcome: Research Gaps and Practice Priorities; Progress in Palliative Care	(i) Opinion piece (ii) The purpose of this paper was to address two important gaps in caregivers' understanding and experiences of the anticipation/preparing for the death of whom they are caring for and the relationship between pre death and post death distress	Australia	(i) There are methodological limitations on what we know about caregiving such as the preparation of caregivers is normally assumed and not assessed directly, postdeath adjustment needs to be assessed differently from quantitative/short period of time, lack of adequate comparison groups in longitudinal studies, and issues in recruiting and retaining participants (ii) Recommendations include more research into caregivers' understanding of preparing for death and relationship between grief and distress before and after death	Research methodology and caregiving	CINAHL

[17]	Nedjat-Haiem et al.; Advocacy at the End of Life: Meeting the Needs of Vulnerable Latino Patients; Social Work in Health Care	(i) Qualitative (ii) Purpose was to explore healthcare providers' experiences with advocacy in working with vulnerable Latinos	United States	(i) Gaps in patient care especially for Latino patients trying to access care within the public healthcare system (ii) Providers looked to advocacy to improve patient care (iii) Need for non-judgemental support, someone who will listen to them and put their needs above that of the organization (iv) Need to present information in a culturally appropriate way	Clinical and system access to care	PubMed

[18]	Higginson; Research Challenges in Palliative Care; BMJ Supportive and Palliative Care	(i) Editorial (ii) The purpose of this paper was to discuss the state of palliative care and research challenges in the field	United Kingdom	(i) From the palliative and end-of-life care priority setting partnership study, they found that some priorities in palliative care are how to best provide care outside of working hours, crisis management, and the ability to help people stay in their place of choice and how to improve access to palliative care (ii) More evidence is needed to support the practical elements of working in palliative care (iii) In a paper by Gott et al. (2013), some challenges faced by staff are communicating palliative care needs	Clinical and research methodology	Hand search
[19]	Murray; Assessing and Addressing Research-Practice Gaps in Practitioners' Provision of Decision Support for Terminally-Ill Patients Considering the Place of their End-of-Life Care	(i) Qualitative and quantitative (ii) The purpose of this study was to address barriers to providing decision support for place of end-of-life care and to describe nurses' perceptions on this topic and to evaluate an educational intervention designed to strengthen quality of practitioners' decision support	United States	(i) One review highlighted gaps in the completeness, balance, and accuracy of information presented in the patient decision aids (PtDAs), which were evaluated (Feldman-Stewart et al., 2006) (ii) Other evidence indicated gaps in nurses' awareness of patient decision-making needs (iii) Participants stated gaps in their education and support systems limited their ability to meet patients' decision-making needs, and they recommend interactive programs with the opportunity for skill development and practice to address this gap (iv) Need for development of tools and processes in combination with provider training to mediate the gap between desired and actual decision making of patients (v) Gaps in education and support systems limit the ability to meet patients' decision-making (vi) Targeted education and practical support by employers were identified as strategies to narrow the skills/confidence gap	System access to care and clinical	Ageline

[20]	Unroe and Meier; Research Priorities in Geriatric Palliative Care: Policy Initiatives; Journal of Palliative Medicine	(i) Review paper (ii) Purpose of this paper was to identify gaps in policy-relevant research and to highlight the disconnect in translating geriatric palliative care models/principles into practice	United States	(i) Targeting a population that will benefit from a given intervention (ii) Expanded evidence base is needed to show the extent to which geriatric palliative care models can improve quality (iii) Gaps in quality measures for the care of patients, for example, many have multiple conditions and do not fall easily into categories which focus on single diseases (iv) Shortages of providers trained in caring for older patients with palliative care needs	Research methodology and clinical	PubMed
[21]	Hay et al.; Developing a Practice-Based Research Agenda for Grief and Bereavement Care; Death Studies	(i) Quantitative (ii) The purpose of this study was to identify priorities for grief and bereavement research	Australia	(i) Gaps exist in understanding growth, resilience, and recovery during the grief process and destigmatizing grief and understanding grief in younger individuals such as children and adolescents and grief from a traumatic death (ii) Gaps also exist in differentiating grief from other mental illnesses and also recognizing grief to set up the correct counselling services (iii) Gaps exist in understanding end-of-life care grief on family caregivers and the impact caregiving has on grief (iv) Practitioners expressed a desire for more research into end-of-life decision making and the relationship between advance care planning and advance directives and grief	Clinical and caregiving	Hand search

[22]	Morrison; Health Care System Factors Affecting End-of-Life Care; Journal of Palliative Medicine	(i) Review paper (ii) The purpose of this paper was to identify needs and gaps of patients and family members	United States	Our current reimbursement system fails to address many of the needs to address patients with serious and chronic illness	System access to care	PubMed

[23]	Canadian Cancer Society; Right to Care: Palliative Care for All Canadians	(i) Report (ii) The purpose of this report was to highlight major gaps in care and existing barriers in palliative care	Canada	(i) Gaps in location of death, use of acute care before death, and referrals to formal palliative care (ii) Surveillance data on palliative care remain sparse and inconsistently collected, leaving gaps in our knowledge (iii) Gaps are lack of common standards or frameworks, insufficient and inconsistent data collection, insufficient education for healthcare providers, lack of support for caregivers, inadequate investment in palliative care, and misunderstanding of palliative care (iv) Recommendations include more support for caregivers and patients and families overall; making palliative care accessible to all; setting guidelines or a minimum standard such as bed numbers or healthcare professionals needed and better education for healthcare workers	System access to care, clinical, research methodology, and caregiving	Hand search
[24]	Walshe; Palliative Care Research: Has It Come of Age?; Palliative Medicine	(i) Editorial (ii) The purpose of this editorial was to discuss how to overcome research challenges in palliative care	United Kingdom	(i) Some methodological challenges that exist in palliative care research are continuing implementation, active recruitment that is also precise, randomization, and economic evaluation that goes beyond looking at saving money (ii) Researchers should refine and develop practice recommendations through critical adoption and feeding back experience	Research methodology	Hand search

[25]	Ritchie and Zulman; Research Priorities in Geriatric Palliative Care: Multimorbidity; Journal of Palliative Medicine	(i) Review paper (ii) The purpose of this paper was to highlight gaps in geriatric palliative care	United States	(i) One gap highlighted was the exclusion of older adults and multimorbid individuals from clinical trials (ii) Another gap is what conditions count/can be excluded or included in a multimorbidity index and need for guidance regarding the illness burden that is generated by common combinations of conditions, interactions among those conditions, and more standardized approaches to multimorbidity assessment	Research methodology and system access to care	PubMed

[26]	California Healthcare Foundation; Racial, Cultural and Ethnic Factors Affecting the Quality of End-of-Life Care in California: Findings and Recommendations	(i) Report (ii) The purpose of this report was to examine end-of-life care delivery for Californians and to understand the impact of race, culture, and ethnicity on the provision of this care	United States	(i) Few studies have addressed situations for decision-making in sudden trajectory situations (ii) There is also a need for cultural sensitivity and competence in end-of-life care	Clinical, caregiving, and system access to care	Hand search

[27]	White and Coyne; Nurses' Perceptions of Educational Gaps in Delivering End-of-Life Care; Oncology Nursing Forum	(i) Quantitative (ii) The purpose of this study was to assess competencies viewed as most important in end-of-life care and to describe what is associated with these competencies	United States	(i) 25% of participants did not believe that they were prepared to care for dying patients (ii) Nurses perceived a widening gap in quantity and quality of continuing education (iii) Recommendations include to continually define and improve symptom management programs in nursing education	Clinical	PubMed
[28]	Hudson et al.; Psychological and Social Profile of Family Caregivers on Commencement of Palliative Care; Journal of Pain and Symptom Management	(i) Experimental (ii) The purpose of this study was to analyze the psychological and social profile of palliative caregivers at the beginning of receiving palliative care services	United States	(i) Caregivers reported high levels of caregiver esteem (ii) Close to half of participants reported an anxiety or depressive disorder (iii) Preloss grief, anxiety, depression, and demoralization were found to be highly correlated with psychological distress (iv) Need for a better understanding of prevalence of anxiety/depression in the caregiving population (v) Caregivers who had a probable anxiety and/or depressive disorder also reported higher levels of preloss grief than caregivers without these disorders. Caregivers who were females, who lived with the patient, had poorer health, and who were caring for a spouse also reported higher levels of preloss grief compared with caregivers who were males, who were not living with the patient, had better health, and who were caring for a parent, respectively (vi) Need for more research in demoralization among family caregivers and to explore the potential benefits of meaning-based therapeutic approaches (vii) More research needed into better interventions	Clinical and caregiving	PubMed

[29]	Garcia; Systematic Review of the Literature on Why There is Hospice Underutilization	(i) Systematic review (ii) The purpose of this review was to focus on the under usage of hospice services and to highlight needs and gaps in palliative care	United States	(i) Research to practice gap (ii) Lack of community education gives hospice poor reputation, and the definition of hospice care is poorly understood (iii) Some recommendations include being able to organize healthcare delivery to create efficient, high-quality, and cost-effective care; understanding operational effectiveness and the quality of patient outcomes; and creating more partnerships between healthcare agencies such as a nursing home and hospitals in order to create care that is patient-centred	Clinical and caregiving	ProQuest Dissertations and Theses
[30]	Herr et al.; Assessing and Treating Pain in Hospices: Current State of Evidence-Based Practices; Journal of Pain and Symptom Management	(i) Experimental study (ii) Purpose of this study was to report on provider evidence-based assessment and treatment practices for older adults with cancer in community-based hospital settings	United States	(i) Practice gaps in completing comprehensive assessment, reviewing pain treatment plan during reassessment, and monitoring side effects (ii) Assessment tools needed for diverse cultural groups (iii) Implications of not having a comprehensive assessment include incomplete records, missing important information, which could limit knowledge of other healthcare practitioners accessing files (iv) Recommendations include strategies beyond education that are more intensive	Clinical	PubMed

[31]	McEvenue; Palliative Care in Long-Term Care: A Multi-Methods Approach to Assessing Quality	(i) Qualitative (ii) Purpose of this study was to define high quality hospice palliative care service delivery through the exploration of approaches taken to provide palliative care at Veterans Affairs Canada (VAC) facilities across Canada	Canada	(i) There is little research available relevant to defining a quality hospice palliative care program in the long-term care setting (ii) Many also felt that, in comparison with physical needs, it was much more difficult to meet the emotional needs of residents, and that improvements in this area were still necessary. As well, meeting the emotional needs of the family was an ongoing challenge in the long-term care setting (iii) Lack of support in family caregivers (iv) Bereavement care for families was lacking at some locations used in the study and is a notable gap in services (v) Recommendations include smaller patient groups to ensure practitioners can form a relationship with the patients; a wide range of training in minority cultures for spiritual providers; a support team available at all HPC teams and modifications to what is looked at when facilities review budgets such as resident accommodations	Research methodology, clinical, and caregiving	ProQuest Dissertations and Theses

[32]	Byrne, Upton, and Townsend; Mind the Gap: A Step Forward in Supporting Hospice-Based Research; BMJ Supportive and Palliative Care	(i) Editorial, highlighting gaps and future steps for hospice-based research (ii) Proposal of a toolkit to help guide organizations and define roles and responsibilities in terms of research studies	United Kingdom	(i) Applying research to care not just methodologically (ii) A need to follow patients across settings and times (iii) There is a gap in hospice organizations' understanding of research practicalities and procedures	Research methodology	Hand search
[33]	Six Nations of the Grand River Territory; Translating Indigenous Knowledge into Palliative Care Policy and Practice: Dissemination Meeting Report	(i) Report (ii) The purpose of this work was to identify needs in end-of-life care for First Nations communities in Canada	Canada	(i) Need to create formal agreements and understandings along with policy implementation (ii) Implementing culturally safe and relevant training programs for healthcare providers	Clinical and system access to care	Hand search

[34]	Holroyd-Leduc et al.; Stakeholder Meeting: Integrated Knowledge Translation Approach to Address the Caregiver Support Gap; Canadian Journal on Aging	(i) Report (ii) Purpose was to review current research and conduct dialogue and identify gaps	Canada	(i) Gaps include the challenges of identifying the population and support needed by caregivers who provide end-of-life care (ii) Recommendations include using results from this meeting to inform future research in caregiver supports and policies to support caregivers	Caregiving and research methodology	PubMed

[35]	Kelley et al; Leveraging the Health and Retirement Study to Advance Palliative Care Research; Journal of Palliative Medicine	(i) Quantitative (ii) The purpose of this study was to describe how the health and retirement study could be used for improving palliative care research	United States	(i) There is no unifying definition for a target population for palliative care services (ii) There is little focus on the needs and outcomes of family caregivers during palliative or end-of-life phases or on the care preferences of patients (iii) There is a need to create and test the use of the Health and Retirement Study's measures for examining quality of care for nursing home residents with serious illness (iv) More communication training for providers and interventions to elicit patient preferences and healthcare systems design to record preferences and reconcile care plans and quality metrics with these stated goals (v) Need to strengthen post death interviews (vi) Lack of evidence on symptom burden and symptom management. Some recommendations to address this are to conduct research that focuses specifically on symptoms	Caregiving, research methodology, and clinical	Hand search

[36]	Canadian Institute for Health Information; Health Care Use at the End of Life in Western Canada	(i) Report (ii) The purpose of this report was to analyze statistics and data pertaining to healthcare service use during the end of life	Canada	(i) There is a lack of standardized key data elements on death certificates, hospital discharge abstracts, and provincial drug plans (ii) There is a need for qualitative and quantitative studies centering around healthcare preferences, quality of care, and innovative models for delivering cost-effective end-of-life care and a need for studies of the appropriateness of dying in hospital, access to community-based supportive care resources, and the adequacy of symptom management at the end of life	Research methodology and system access to care	Hand search

## Data Availability

The dataset generated (data extraction table) will be available as part of the online submission of this published article. All other datasets and/or data analyzed during the current scoping review are available from the corresponding author upon reasonable request.

## Abbreviations

ACP: Advance care planning
CHPCA: Canadian Hospice Palliative Care Association
CIHR: Canadian Institutes of Health Research
CPCA: Canadian Palliative Care Association (now: CHPCA)
EoL: End-of-life care
HPC: Hospice and palliative care
MAiD: Medical assistance in dying
MDS: Minimum data set
QELCCC: Quality End-of-Life Care Coalition of Canada.
